# Investigation of the atypical *FBXW7* mutation spectrum in human tumours by conditional expression of a heterozygous propellor tip missense allele in the mouse intestines

**DOI:** 10.1136/gutjnl-2013-304719

**Published:** 2013-05-15

**Authors:** Hayley Davis, Annabelle Lewis, Axel Behrens, Ian Tomlinson

**Affiliations:** 1 Molecular and Population Genetics Laboratory, Wellcome Trust Centre for Human Genetics, Oxford University, Oxford, UK; 2 Mammalian Genetics Laboratory, London Research Institute, Cancer Research UK, London, UK

**Keywords:** Cancer, Cancer Genetics, Colon Carcinogenesis, Colorectal Cancer

## Abstract

**Objective:**

*FBXW7* encodes the substrate recognition component of a ubiquitin ligase that degrades targets such as Notch1, c-Jun, c-Myc and cyclin E. *FBXW7* mutations occur in several tumour types, including colorectal cancers. The *FBXW7* mutation spectrum in cancers is unusual. Some tumours have biallelic loss of function mutations but most have monoallelic missense mutations involving specific arginine residues at β-propellor tips involved in substrate recognition.

**Design:**

FBXW7 functional studies have generally used null systems. In order to analyse the most common mutations in human tumours, we created a *Fbxw7^fl(R482Q)^*^/+^ mouse and conditionally expressed this mutation in the intestines using *Vill*-Cre. We compared these mice with heterozygous null (*Fbxw7*^+/−^) mutants.

**Results:**

A few sizeable intestinal adenomas occurred in approximately 30% of R482Q/+ and *Fbxw7*^+/−^ mice at age >300 days. Breeding the R482Q allele onto *Apc* mutant backgrounds led to accelerated morbidity and increased polyp numbers and size. Within the small bowel, polyp distribution was shifted proximally. Elevated levels of two particular Fbxw7 substrates, Klf5 and Tgif1, were found in normal intestine and adenomas of R482Q/+, R482Q/R482Q and *Fbxw7*^−/−^ mice, but not *Fbxw7*^+/−^ animals. On the *Apc* mutant background, *Fbxw7*^+/−^ mutants had a phenotype intermediate between *Fbxw7* wild-type and R482Q/+ mice.

**Conclusions:**

Heterozygous *Fbxw7* propellor tip (R482Q) mutations promote intestinal tumorigenesis on an *Apc* mutant background. Klf5 and Tgif1 are strong candidates for mediating this effect. Although heterozygous null *Fbxw7* mutations also promote tumour growth, these have a weaker effect than R482Q. These findings explain the *FBXW7* mutation spectrum found in human cancers, and emphasise the need for animal models faithfully to reflect human disease.

**Significance of this study**

**What is already known about this subject?**

- *FBXW7* is commonly mutated in tumours of diverse origins, including colorectal cancer.
- *FBXW7* is classed as a tumour suppressor, but has an unusual mutation spectrum whereby biallelic, simple loss-of-function mutations are rare; instead, most mutations are monoallelic missense changes involving specific arginine residues at β-sheet propellor tips that allow the FBXW7 protein to recognise its substrates.
- To date, mouse models carry *Fbxw7* null alleles, but these do not faithfully recapitulate the mutations most commonly present in human cancers.

**What are the new findings?**

- Conditional expression of a heterozygous propellor tip missense *Fbxw7* allele in the mouse intestines causes tumorigenesis.
- The mutation causes elevated levels not of classical Fbxw7 substrates such as c-Jun, but of Klf5 and Tgif1, in both normal intestines and adenomas.
- On an *Apc* mutant background, heterozygous null *Fbxw7* mutations promote tumour growth, but have a much weaker effect than heterozygous propellor tip mutants.
- Propellor tip mutations most likely act as dominant negative, loss-of-function alleles that provide sufficient derangement for tumorigenesis and they are found commonly because they require only a single ‘hit’.

**How might it impact on clinical practice in the foreseeable future?**

- Use of specific animal models can explain the in vivo mutation spectrum of cancer genes.
- Genetically targeted therapies need to take into account the specific mutations in cancer and how they act.
- FBXW7 substrates exhibit tissue specificity; here, TGIF1 and KLF5 have been identified as the strongest candidate substrates for therapeutic intervention.

## Objective

*FBXW7* (F-box and WD40 repeat domain containing 7, also known as *FBW7*, *hCDC4*), a tumour suppressor gene, is mutated in many human malignancies.^1^ *FBXW7* encodes the substrate recognition component of a Skp, Cullin, F-box-containing (SCF)-E3 ubiquitin ligase complex and negatively regulates multiple proteins with established roles in the control of cell division and growth, including cyclin E, c-Jun, c-Myc, mTor and Notch. There are several excellent reviews of the growing knowledge of FBXW7.^2^^–^5

As it became apparent that FBXW7 regulates many oncoproteins, it was hypothesised that mutations in *FBXW7* may cause tumorigenesis. *FBXW7* mutations were initially identified by Spruck *et al* in ovarian and breast cancer cell lines.^6^ ^7^ Since then many studies have assessed *FBXW7* mutation status in a range of cancer types, including both solid tumours and haematological neoplasms. A study by Akhoondi *et al*^8^ significantly contributed to this collection, as extensive genetic screening of over 500 primary tumours of diverse tissue origins was carried out, identifying mutations in *FBXW7* in 6% of tumours. Variations in mutation frequencies among these tissues were seen, with the highest frequencies found in lesions from the bile duct, endometrium, blood and colorectrum.

FBXW7 has several important domains, including WD40^9^ repeats that form an eight-bladed, barrel-shaped β-propellor (see online supplementary figure S1A) which provides a binding pocket for substrates.^10^ ^11^ Critical arginine residues at the apex of the propellors directly interact with ‘destruction recognition’ (CDC4 phosphodegron, CPD) sequences in substrates.^10^ ^11^ The Catalogue of Somatic Mutations in Cancer (COSMIC) database reports 496 mutations in *FBXW7* across all tissue types; 53% of these are missense changes affecting arginine residues 465, 479 and 505 that lie at the β-propellor tips which interact with FBXW7 substrates, with the majority of these propellor tip mutations being monoallelic.

*FBXW7* mutations have been investigated thoroughly in colorectal tumours.^12^^–^14 In colorectal cancer, 189 mutations have been reported, of which 44% are missense mutations at amino acids 465, 479 and 505. We have analysed The Cancer Genome Atlas (TCGA) set of 226 colorectal cancers and find no evidence that arginine propellor tip missense mutations are associated with clinicopathological variables, including gender, age of presentation and cancer stage (details not shown). Non-propellor tip mutations are found more frequently in hypermutated cases (p=0.04), suggesting that some of these mutations are background changes.

Loss of heterozygosity at *FBXW7* is observed infrequently, especially for WD40 propellor tip mutations, and few tumours carry biallelic *FBXW7* mutations predicted to abolish protein function.^13^ ^15^ These data suggest that *FBXW7* does not function as a standard tumour suppressor gene through the two hit mechanism of inactivation,^16^ and partial loss of FBXW7 function may be sufficient to cause tumorigenesis. We have formalised this suggestion in the ‘just enough’ model^15^ whereby protein-inactivating monoallelic (heterozygous) *FBXW7* mutations provide insufficient functional derangement to drive tumour growth, although they can do so as homozygotes or compound heterozygotes. However, monoallelic propellor tip mutations cause an intermediate level of functional derangement that is sufficient to promote tumorigenesis.

Two groups have previously generated simple knockout mice carrying null *Fbxw7* alleles.^17^ ^18^ In the heterozygous mutant state, these mice appeared normal with no tumour formation up to 1 year of age. However, null homozygotes suffered embryonic lethality at E10.5-11.5 due to compromised cardiovascular development that was attributed to elevated Notch1 expression.^17^ ^18^ Subsequently, conditional *Fbxw7*-null mice were used to study Fbxw7 in T cell,^19^ haematopoietic stem cell^20^ ^21^ and gut lineages.^22^ ^23^ Sancho *et al*^22^ showed that wild-type *Fbxw7* mRNA was highly expressed in the transit amplifying/progenitor cell compartment of the intestines, with a threefold increase in the crypt compared with the villus.^22^ Homozygous *Fbxw7* knockout resulted in impaired intestinal cell differentiation, with a reduction in the number of goblet and Paneth cells. There was an accumulation of intracellular domain of Notch1 (Nicd1) and phosphorylated c-Jun. Heterozygous *Fbxw7* knockout resulted in an accumulation of Nicd1, but not phospho-c-Jun. Notch is a key regulator in the binary decision of transit amplifying cells to differentiate into secretory or absorptive cells and therefore elevated Nicd1 protein levels in *Fbxw7* null cells were reported as the likely cause of the differentiation phenotype observed. Despite the accumulation of actively proliferating progenitor cells in the guts of homozygous null mice, no intestinal tumours were reported up to 11 months of age. Another group^23^ subsequently studied the same conditional *Fbxw7* null mouse, finding a small number of adenomas in the large intestines of homozygous knockout animals at 9–10 months of age. Increased crypt budding and fission, but no polyps, were observed in the small intestine.

As the *Fbxw7* null mutant mice do not faithfully recapitulate the mutations most commonly present in human cancers, we have generated a conditional *Fbxw7* mutant mouse which carries one of the most commonly occurring propellor tip missense mutations. We have used this to determine the consequences of propellor tip mutations, and compared the phenotypic effects of this mutation with those of heterozygous null alleles, both in the normal intestine and in intestinal polyps.

## Design

### Generation and genotyping of R482Q mice

Generation and genotyping of *Fbxw7^fl(R482Q)^*^/+^ mice were described previously^24^ (see online supplementary figure S1B). *Fbxw7^fl(R482Q)^*^/+^ mice were crossed with *Vill*-*Cre* mice^25^ in order to knock-in the R482Q mutation (equivalent of human R479Q) in the intestine. These mice shall be referred to as R482Q/+ in the heterozygous state. Conditional *Fbxw7* null animals^22^ were crossed with *Vill-Cre* animals; these will be referred to as *Fbxw7*^+/−^ in the heterozygous state. Two types of *Apc* mutant mice, *Apc^Min^*^/+^ (Min)^26^ and *Apc^1322T^*^/+^ (1322T),^27^ were also used in this study. All procedures were carried out in accordance with Home Office UK regulations and the Animals (Scientific Procedures) Act 1986. All mice were housed at London Research Institute, Clare Hall Laboratories, Cancer Research UK or Functional Genomics Facility, Wellcome Trust Centre for Human Genetics, Oxford University. All strains used in this study were maintained on the C57Bl/6J background for ≥6 generations.

### Tissue preparation and histology

Mice were sacrificed when showing symptoms of intestinal polyps (anaemia, hunching) by cervical dislocation. The intestinal tract was removed immediately and divided into small intestine (proximal/SB1, middle/SB2 and distal/SB3) and large intestine. The intestines were opened longitudinally, using a gut preparation apparatus,^28^ washed in phosphate buffered saline (PBS) and fixed overnight in 10% neutral buffered formalin. For scoring of polyps, gut preparations were stained with 0.2% methylene blue for 10 s, washed in PBS for 20 min and polyps were counted/measured using a dissecting microscope at 3× magnification. Fixed specimens were embedded and haematoxylin–eosin stained following standard protocols.

### Western blotting

For intestinal tissue, isolation of epithelial cells was carried out. A 2 cm long piece of intestine was opened longitudinally and incubated in PBS supplemented with 30 mM EDTA for 2 h with agitation at 4°C. Shed epithelial tissue was pelleted and lysed in RIPA buffer with the addition of protease inhibitors (Complete Mini, Roche) on ice for 30 min. Nuclear fractions were generated as described previously.^24^ Lysates were quantified using the BCA assay (Thermo Scientific). Western blotting was performed following standard methods using the following antibodies: Cyclin E (sc-481, Santa Cruz), c-Jun (sc-44, Santa Cruz), c-Myc (sc-47694, Santa Cruz), p-c-Myc (Thr58/Ser62) (sc-8000R, Santa Cruz), Klf5 (ab24331, abcam), Tgif1 (H-171) (sc-9084, abcam), activated Notch 1 (Nicd1) (ab8925, Abcam), Lamin B1 (H-90 sc-20682, Santa Cruz), FLAG (F1804, Sigma-Aldrich) and c-Myc epitope tag (S1826, Clontech). Bands were quantified using pixel intensity normalised to loading control (ImageJ).

### Immunohistochemistry

Immunohistochemistry for performed as described previously^24^ using the following antibodies: alkaline phosphatase (ab65834, Abcam), β-catenin (610154, BD), caspase3 (AF835, R and D systems), chromogranin A (ab15160, Abcam), Ki67 (TEC-3, DAKO) and lysozyme (EC3.2.1.17, DAKO). To assess proliferation, the proportion of Ki67 positive cells per crypt was quantified in 200 randomly selected crypts from SB3. To assess apoptosis, the numbers of positive caspase3 cells per crypt–villus unit in SB1, SB2 and SB3 were counted for 100 crypt–villus units.

### Co-immunoprecipitation of tagged *Fbxw7* plasmids

Murine wild-type and R482Q Fbxw7 coding region cDNAs were cloned into pCMV Myc and FLAG tagged mammalian expression plasmids, respectively (Clontech). Plasmids were transiently transfected into RKO cells and co-immunoprecipitated using protein G Dynabeads (Invitrogen) and 2 μg of Myc antibody (S1826 Clontech) and anti-FLAG M2 magnetic beads (Sigma-Aldrich) for the Myc and FLAG co-immunoprecipitation, respectively.

### Expression of exogenous wild-type FBXW7 in colorectal cancer cell lines

The FBXW7 overexpression plasmid, p3xFLAG-Myc-CMV-24, containing human FBXW7α isoform (pFLAG FBXW7α) was kindly provided by Bruce Clurman (Fred Hutchinson Cancer Research Center, Seattle). The same vector lacking the FBXW7α insert was used as an empty vector control (pEV).

### shRNA knockdown of *FBXW7*

pLKO.1 short hairpin RNA (shRNA) lentiviral clones TRCN0000006556 (6) and TRCN0000006557 (7) (Open Biosystems) were used to knockdown *FBXW7* expression. Lentiviral particles were packaged following standard protocols and titrated using the Lenti-X p24 Rapid Titre Kit (Clontech). LOVO cells were transduced with 100 ng of packaged virus. Efficient knockdown of *FBXW7* mRNA (∼75%) was detected by quantitative real–time PCR using TaqMan probe Hs00217794_ml (Applied Biosystems).

### Additional methods

Additional methods are provided in the online supplementary data.

## Results

### Generation of intestine-specific R482Q and *Fbxw7* null mutant mice

To ascertain whether *Fbxw7* propellor tip mutations could induce intestinal tumorigenesis, *Fbxw7^fl(R482Q)^*^/+^ mice were bred with *Vill-Cre*^25^ animals to give *Vill-Cre*;*Fbxw7^R482Q^*^/+^ (R482Q/+) mice. These mice express Cre recombinase in all cell types of the intestinal epithelium^29^ and we confirmed knock-in of the R482Q mutation along the entire intestine (see online supplementary figure S1C, D). Similarly, for our comparison group, we crossed conditional *Fbxw7^fl(Δex5)^*^/+^ null animals with *Vill*-*Cre* mice to give *Vill-Cre*;*Fbxw7^fl(Δex5)^*^/+^ (*Fbxw7^+/−^*) animals and confirmed efficient Cre-mediated recombination as above.

### Normal intestines of *Fbxw7* mutant mice show subtle alterations in cell lineages and elevated levels of apoptosis

To ascertain whether molecular defects were present in normal intestinal tissue in young *Fbxw7* mutant mice, we examined a range of cell lineage markers. There were no significant differences in crypt size or the positions of specialised cell types among mice (details not shown). The number of goblet cells per villus, which varied from seven to 11 across all mice (see online supplementary figure S2), showed an R482Q dosage-dependent reduction in numbers compared with wild-type (p<0.001, non-parametric trend test), in agreement with previous findings in *Fbxw7^+/−^* and *Fbxw7^−/−^* animals^22^ that we also replicated here (details not shown). There were no significant differences in any of the other cell lineages (see online supplementary figure S2) and no change in the size of the stem cell compartment, as assessed using *Lgr5* and *Olfm4* mRNA expression.

As many Fbxw7 substrates are critical proteins in proliferation and apoptosis, these processes were assessed in morphologically normal intestinal tissue from *Fbxw7* mutant mice and controls. No significant difference in proliferation was observed (see online supplementary figure S3A, B). However, there were significant dosage-dependent increases in apoptosis both in R482Q/+ and R482Q/R482Q animals (p<0.001, non-parametric trend test), and in *Fbxw7^+/−^* and *Fbxw7^−/−^* animals (p=0.010) compared with wild-type (see online supplementary figure S3A, C). There was no significant difference in apoptosis between the R482Q and Fbxw7 null alleles (p=0.138, ANOVA).

### Normal intestine from R482Q/+ mice, but not *Fbxw7^+/−^* animals, shows elevated levels of Fbxw7 substrates Klf5 and Tgif1

**Figure 1:**
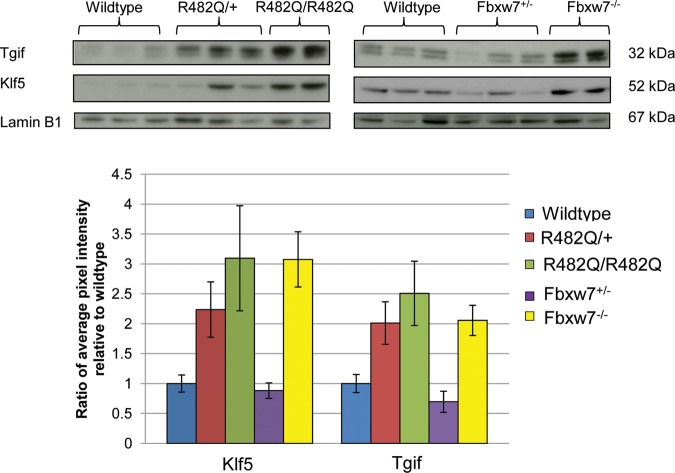
Elevated Klf5 and Tgif1 protein in normal intestine of R482Q mice. Representative western blots are shown. Tgif1 protein was detected as a double band, as previously reported, with the upper band representing the hyperphosphorylated form.^40^ The bar chart shows quantitation of western blots derived from the ratio of pixel intensity for each test protein normalised to its Lamin B1 control. Mean protein levels in mutant mice are displayed in the chart relative to the level in wild-type mice. Western blots for each antibody were performed in at least two independent experiments, each with two technical replicates.

As Fbxw7 regulates a plethora of substrates, we selected for analysis those known to be oncoproteins or involved in intestinal development or homoeostasis. We found no significant difference in the protein levels of Aurora A, Nicd1, c-Myc, p-c-Myc or c-Jun between wild-type, R482Q/+ and R482Q/R482Q mice (see online supplementary figure S4). Elevated levels of cyclin E were found in normal R482Q/R482Q tissue compared with wild-type controls (p=0.003, t test), although no significant increase in protein expression was seen in R482Q/+ tissue (figure 1). Two substrates, Tgif1 and Klf5, showed elevated levels in both R482Q/+ and R482Q/R482Q animals (for Tgif1, p=0.003 and p=0.001, respectively; for Klf5, p=0.036 and p=0.010, respectively; t test). The trend towards increased substrate levels with R482Q dosage was significant in both cases (for Tgif1, p=0.002 and for Klf5, p=0.048, non-parametric trend test). However, there were no significant differences in Tgif1 or Klf5 levels between R482Q/+ and R482Q/R482Q mice (p=0.547 and p=0.567, respectively, t test).

Fbxw7 substrates were then assessed in *Fbxw7^+/−^* and *Fbxw7^−/−^* mice (figure 1, see online supplementary figure S4). For both Tgif1 and Klf5, we found no increase in protein levels over wild-type in *Fbxw7^+/−^* animals (for Tgif1, p=0.440 and for Klf5, p=0.433, t test). However, significant increases were found in *Fbxw7^−/−^* animals (for Tgif1, p=0.002 and for Klf5, p=0.001, t test). No dosage-dependent elevation in levels was found (p=0.064 for Tgif1 and p=0.060 for Klf5, non-parametric trend test), and there were significant differences in Tgif1 and Klf5 levels between *Fbxw7^+/−^* and *Fbxw7^−/−^* mice (p=0.007 and p=0.008, respectively, t test).

Finally, we compared Tgif1 and Klf5 across the R482Q- and *Fbxw7^−^* mice. The level of Tgif1 protein in R482Q/+ tissue was significantly higher than in Fbxw7^+/−^ tissue (p=0.026, t test). The level of Klf5 was similarly higher in R482Q/+, although this did not reach formal significance (p=0.105). There were no significant differences in Tgif1 and Klf5 levels among R482Q/+, R482Q/R482Q and *Fbxw7^−/−^* tissues (p>0.45 in all cases, t test).

### Intestinal tumours occur in aged R482Q/+ and *Fbxw7^+/−^* mice

R482Q/+ mice developed no intestinal polyps or other gross defects up to 215 days of age (n=8). However, a R482Q/+ mouse showed signs of distress at 409 days old and was found to carry a large polyp in the small intestine (see online supplementary figure S5), which on further histological analysis was identified as an (invasive) adenocarcinoma (see online supplementary figure S5D). We then aged a cohort of 41 R482Q/+ mice to >300 days old. Twelve (29%) mice exhibited a low number (1–3) of small intestinal adenomas, most of which (18/27, 67%) were >3 mm in diameter. Adenomas were located in all parts of the small intestine. Twelve wild-type mice of a similar age showed no intestinal phenotype (p=0.048, Fisher’s exact test).

A cohort of 13 *Fbxw7^+/−^* mice aged >300 days was analysed and two (15%) displayed a single intestinal polyp. Both of these polyps were >3 mm in diameter, located in SB2 and SB3, and were non-invasive. There was no significant difference in the proportion of mice with polyps between R482Q/+ and *Fbxw7^+/−^* animals (p=0.377, Fisher's exact test).

### Adenomatous polyps from R482Q/+ mice exhibit activation of the Wnt signalling pathway

**Figure 2:**
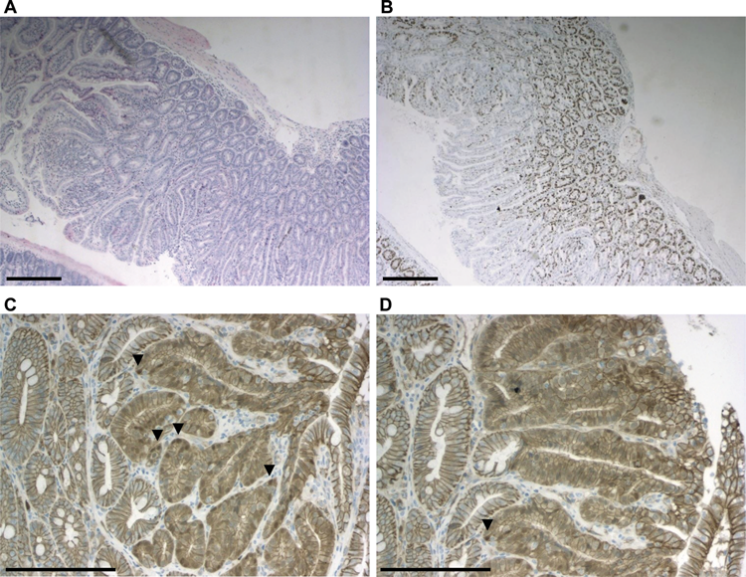
Proliferation and Wnt activation in representative R482Q/+ polyps shown by immunohistochemistry. (A) haematoxylin–eosin stained section of polyp—scale bars 300 μm. (B) Ki67 expression in polyp—scale bars 300 μm. (C, D) β-Catenin expression—scale bars 150 μm; arrowheads highlight nuclear staining.

The late age of presentation of tumours and incomplete penetrance of phenotype in R482Q/+ mice suggested that accumulation of additional mutations was necessary for tumour development. As the majority of intestinal polyps acquire activated Wnt signalling, we assessed this pathway by investigating localisation of β-catenin protein using immunohistochemistry (figure 2). In general, cytoplasmic and low level nuclear β-catenin expression was observed, comparable with polyps from 1322T mice.^27^ We then screened 11 R482Q/+ polyps for somatic mutations in *Apc* (exon 15), *Ctnnb1* (exon 2), *Tp53* (exons 5-8) and *Kras* (exons 1–2). Two *Ctnnb1* p.Thr41Ile mutations were found (see online supplementary figure S6) in polyps from different mice. This is a known driver mutation in human colorectal tumours. We found no loss of heterozygosity of *Fbxw7* in R482Q/+ tumours (n=4). To test for other chromosomal deletions or gains, array comparative genomic hybridisation was carried out on DNA extracted from the R482Q/+ adenocarcinoma, but no copy number variants were identified.

### R482Q enhances intestinal polyposis in *Apc* mutant mice

Multiple mutations are necessary for carcinogenesis, and as *APC* mutations occur in a large majority of human colorectal cancers, *FBXW7* mutations are found mostly in the context of *APC* mutant alleles. To address whether Fbxw7 propellor tip mutant protein could promote the development of intestinal polyposis initiated by *Apc* mutations, R482Q mice were bred with two *Apc* mutants, Min^26^ and 1322T.^27^ R482Q/+; *Apc* compound mutant (test) and *Fbxw7* wild-type; *Apc* mutant (control) mice were aged until test mice were symptomatic. Control littermates were then sacrificed alongside the test mice. Although multiple adenomatous polyps were present in all animals, invasive tumours were not detected in either R482Q/+;Min or R482Q/+;1322T mice—this may be a result of the shortened lifespan of mice due to tumour burden, which prevents the accumulation of the somatic mutations necessary for tumour progression.

R482Q/+;Min mice were sacrificed at 87–111 days (see online supplementary figure S7). There was a significantly greater number of tumours in the R482Q/+;Min mice than the Min mice (p=0.002, t test; see online supplementary figure S7A). Both types of mouse displayed most polyps in SB3 (see online supplementary figure S7B). Polyps also tended to be larger in the double mutant animals (see online supplementary figure S7C); in Min animals, the majority of polyps were <1 mm in diameter, whereas in R482Q/+;Min compound mice, most were 1–2 mm in diameter (p=0.0155, t=test).

The equivalent comparison was then performed with 1322T rather than Min mice. R482Q/+;1322T animals showed signs of an intestinal phenotype at 71–127 days. There was a modest, non-significant increase in total tumour numbers in R482Q/+;1322T mice compared with 1322T controls (p=0.769, t test; see online supplementary figure S7A). R482Q/+;1322T polyps showed the same distribution as 1322T polyps, with the heaviest burden in SB1 (see online supplementary figure S7B), and the presence of the R482Q mutation had no significant impact on tumour size (p=0.651; see online supplementary figure S7C).

A combined stratified analysis of the effects of R482Q in both Min and 1322T mice was then performed. This showed that total number (p=0.0075, ANOVA) and size (p=0.011, logistic regression) of polyps was increased by the presence of the R482Q mutation (see online supplementary figure S7A). Only two R482Q/R482Q; *Apc* mutant mice were available for analysis, but these showed significantly more and larger polyps than *Fbxw7* wild-type mice (p=0.0014 and p=0.012, respectively).

**Figure 3:**
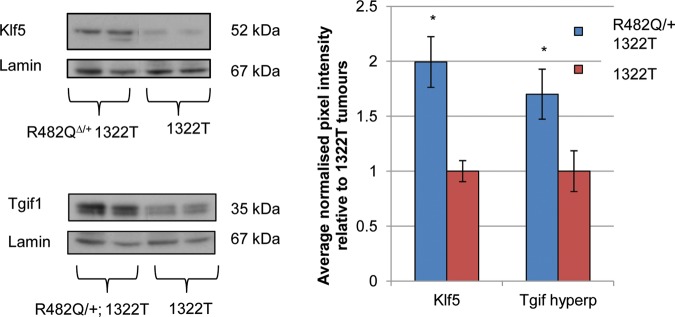
Elevated levels of Klf5 and Tgif1 protein in *R482Q/+*;*Apc* mutant tumours. The graph shows the ratio of average normalised pixel intensities, relative to 1322T tumours. The hyperphosphorylated (hyperp) band of Tgif1 was quantified, consistent with the paradigm that Fbxw7 substrates are phosphorylated prior to ubiquitination by Fbxw7. Results represent two technical replicates.

The levels of selected Fbxw7 substrates were analysed in R482Q/+;1322T and 1322T tumours paired for size and location. There was no significant difference in the levels of c-Jun, Aurora A, Nicd1, cyclin E or p-c-Myc between the two types of mice (p > 0.05 in all cases, t test; see online supplementary figure S8). However, levels of Klf5 and Tgif1 were elevated in R482Q/+;1322T compared with 1322T tumours (p=0.045 and p=0.042, respectively, t test, figure 3), consonant with the findings in normal intestinal epithelium.

### R482Q/+ is a stronger promoter of polyposis than *Fbxw7^+/−^* in *Apc* mutant mice

**Figure 4:**
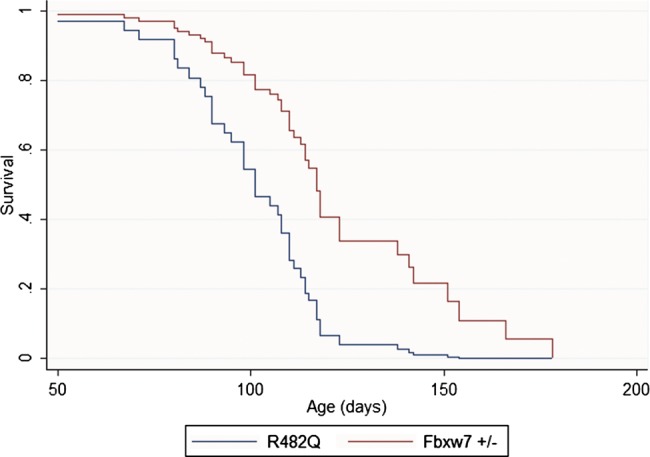
Kaplan–Meier plots of survival of R482Q/+ and *Fbxw7^+/−^* mice on an *Apc* mutant background. Note the more severe disease associated with the R482Q allele. Although not shown, survival of 1322T animals was consistently shorter than that of Min mice for both *Fbxw7* alleles.

Our ‘just enough’ model of *FBXW7* mutations predicts that R482Q heterozygotes have a greater functional effect than null heterozygotes. We therefore compared polyp formation in R482Q/+ and *Fbxw7^+/−^* mice on an *Apc* mutant (Min or 1322T) background using age of developing symptoms as a measure of disease severity. In an analysis stratified by type of *Apc* mutation, we found that R482Q/+ mice presented significantly earlier than *Fbxw7^+/−^* animals (mean 101 vs 122 days, p=0.001, Cox proportional hazards test, figure 4). No significant difference in the number (p=0.127) or size (p=0.661) of polyps between the different *Fbxw7* models at presentation was found, consistent with tumour load being the principal factor determining age of onset of symptoms. The R482Q allele was, however, additionally associated with a shift in polyp distribution to SB1 (39% in R482Q/+ vs 26% in *Fbxw7^+/−^*, p=0.024). Analysis of only two *Fbxw7^−/−^* animals was possible, and their age of presentation was not significantly different from the R482Q/+ animals.

### Propellor tip mutant FBXW7 may act via a dominant negative mechanism

**Figure 5:**
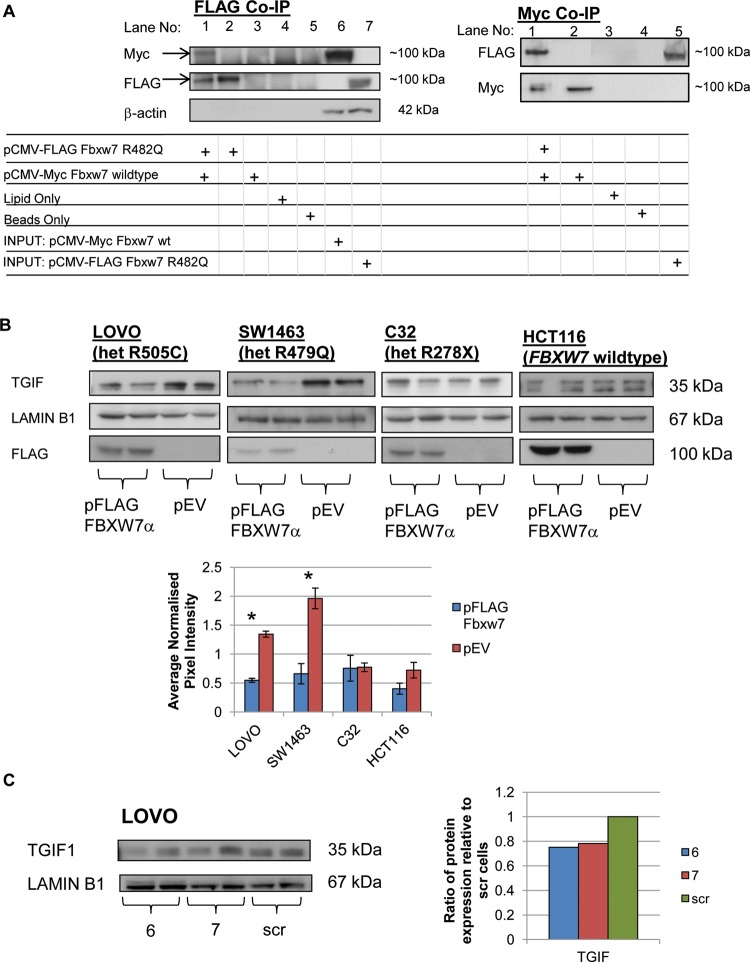
In vitro studies of FBXW7 in colorectal cancer cell lines. (A) Interaction of mouse wild-type and R482Q Fbxw7 protein: co-immunoprecipitation (Co-IP) experiments of tagged Fbxw7 R482Q (FLAG) and wild-type (Myc) plasmids transiently transfected into RKO cells (*FBXW7* wild-type). In FLAG Co-IP, lane 1, cells were transfected with both plasmids and FLAG co-immunoprecipitation performed; western blotting using anti-Myc identifies wild-type Fbxw7 (left, upper), indicating that R482Q mutant and wild-type Fbxw7 proteins interact. Anti-FLAG western blot control is shown (left, lower). In the reverse Co-IP using anti-Myc, western blotting using anti-FLAG similarly identified R482Q mutant Fbxw7 (right, upper). Anti-Myc western blot control is shown (right, lower). These experiments show that Fbxw7 wild-type and R482Q proteins interact. (B) Western blot of the nuclear fraction of a panel of cell lines expressing exogenous wild-type FBXW7 shows significant decreases in TGIF1 levels in propellor tip mutant cells, but not heterozygous null mutant or wild-type cells. The graph shows quantitation of the western blots by measuring average pixel intensities. Error bars represent SEM. *Significant results (LOVO p=0.006, SW1463 p=0.035, t test). Blots probed with anti-FLAG indicate transfection efficiency. KLF5 levels were too low in some of these cell lines for reliable analysis. (C) Knockdown of *FBXW7* using short hairpin (shRNA) in the propellor tip mutant cell line LOVO causes no significant increase in TGIF1 protein levels (p=0.477, t test). Six and seven are results from different FBXW7 shRNA clones. The graph show the ratio of normalised pixel intensities relative to the cells transduced with scrambled shRNA as a control.

Existing evidence showed that propellor tip mutant FBXW7 can act as a dominant negative,^12^ and our own co-immunoprecipitation data supported this by showing that mutant and wild-type Fbxw7 proteins interact (figure 5A). Dominant negative loss of function is therefore the most likely mechanism underlying the more severe phenotype of R482Q/+ than *Fbxw7^+/−^* mice. In further support of this model, we found that overexpression of wild-type FBXW7 causes TGIF1 levels to reduce significantly in colorectal cancer cell lines that carry propellor tip mutations (LOVO, heterozygous R505C and SW1463, heterozygous R479Q) (figure 5B). C32, a control cell line carrying a heterozygous protein truncating mutation of uncertain significance (R278X), showed no decrease in TGIF1 (figure 5B), suggesting that TGIF1 levels in these cells were not significantly influenced by the presence of the monoallelic null allele. Furthermore, shRNA-mediated 75% knockdown of *FBXW7* mRNA in colorectal cancer lines with propellor tip mutations produced no significant increase in TGIF1 levels (figure 5C), suggesting that the monoallelic mutation produced a strong functional effect that could not readily be enhanced in the system used. Overall, while not conclusive on their own, these data support our mouse data showing a significant functional difference between monoallelic propellor tip *Fbxw7* mutations and the heterozygous null genotype.

## Conclusions

We have demonstrated that an *Fbxw7* propellor tip mutation at arginine 482—a site homologous to amino acid 479 that is commonly mutated in human tumours—directly promotes intestinal tumorigenesis in a mouse model. It is likely that this must occur on a background of increased Wnt signalling, such as that provided by germline *Apc* mutations, or by spontaneous somatic mutation of genes such as β-catenin in older animals.

We compared our *Fbxw7^R482Q/+^* (R482Q/+) mice with the *Fbxw7* null mice of Sancho *et al*^22^ bred in the same facility. The principal comparison was to test our hypothesis that the R482Q allele occurs commonly in human tumours because it provides a greater level of functional derangement than a single null *Fbxw7* allele. On an *Apc* mutant background, polyposis developed more rapidly in R482Q than *Fbxw7^+/−^* animals, and there was additionally a relative shift in polyp numbers to SB1, a feature previously associated with more severe disease.^27^ ^30^^–^32

Of the Fbxw7 substrates tested, only Tgif1 and Klf5 protein levels were consistently increased in R482Q mutant normal tissues and in polyps. *Fbxw7^−/−^*—but importantly not *Fbxw7^+/−^*—animals also showed a significant change in Tgif1 and Klf5 expression. KLF5 is a zinc finger family transcription factor that plays a role in multiple cellular processes, including cell cycle regulation, proliferation and angiogenesis.^33^ It is primarily expressed in proliferative regions of the intestinal epithelium,^34^ and most data suggest that it promotes G1–S phase transition.^34^ ^35^ We found no significant changes in proliferation in morphologically normal tissue from R482Q mutant mice but it has been shown^31^ ^36^ that *Klf5^+/−^* mice have a greatly reduced intestinal tumour load. TGIF1 is a negative regulator of the transforming growth factor β (TGF-β) signalling pathway, acting as a co-repressor in Smad2/3 complexes.^37^ Although there is a complex relationship between TGF-β activity and carcinogenesis, TGF-β component inactivation is common in colorectal tumours, and increased TGIF1 may therefore contribute to intestinal tumorigenesis in our mice. To date, however, no direct assessment has been performed of the effects of Tgif1 mutation on intestinal tumours.

We additionally found that cyclin E levels were increased in mice homozygous for the R482Q or null mutation. However, Nicd1 levels were not significantly raised in *Fbxw7^+/−^* or *Fbxw7^−/−^* animals, despite such an increase having been reported previously^22^ ^23^ and being present in our R482Q mutants. There was no good evidence that levels of other known FBXW7 substrates were increased in the mice studied, suggesting that their roles in intestinal tumorigenesis are limited. Current evidence does not suggest that the R482Q mutation affects a qualitatively different range of substrates from the null allele, since Tgif1, Klf5 and cyclin E were all significantly increased in *Fbxw7^−/−^* mice as well as in R482Q mutants. Evidently, it remains possible that unidentified FBXW7 substrates contribute to tumorigenesis and are differentially affected by propellor tip and protein truncating mutations.

In summary, our data support the ‘just enough’ model in showing that the R482Q mutation has a stronger effect on *Apc-*driven intestinal tumorigenesis than a null heterozygote (*Fbxw7^+/−^*) mutation. Tgif1 and Klf5, rather than the classical Fbxw7 substrates, are the best candidate mediators of this differential effect, although the underlying mechanism requires further analysis. Contrary to the ‘just enough’ model, however, the murine *Fbxw7^+/−^* genotype did promote intestinal tumorigenesis, whether alone or on an *Apc* mutant background, whereas we predict that this genotype causes insufficient functional derangement to promote human tumorigenesis. This discrepancy may be a mouse-specific phenomenon resulting from a decreased requirement for ‘second hits’ at *Fbxw7* compared with humans,^38^ as our in vitro data suggest; or it may reflect the fact that heterozygous null human *FBXW7* genotypes do have some, submaximal, tumour promoting effects, perhaps in a continuum fashion.^39^ Overall, our data support the notion that *FBXW7* is not a classical tumour suppressor gene. As propellor tip mutations require only ‘one hit’ and yet have strong enough functional effects for tumorigenesis, they occur more often than the alternative of biallelic protein inactivating mutations.

## Supplementary Material

Web supplement
