# Supplementary material for: Investigation of the atypical FBXW7 mutation spectrum in human tumours by conditional expression of a heterozygous propellor tip missense allele in the mouse intestines
Source: Gut. 2013 May 15;63(5):792–9. doi: 10.1136/gutjnl-2013-304719 (PMC3995284; doi:10.1136/gutjnl-2013-304719)

Supplementary Materials and Methods

cDNA Sequencing

To generate cDNA, RNA was isolated from frozen tissue with the RNeasy minikit (Qiagen), treated with DNase I to degrade residual DNA and complementary DNA was reverse transcribed *in vitro* using the High Capacity cDNA Reverse Transcription Kit (Applied Biosystems). Where required, normal and polyp tissue was microdissected with the laser capture PALM system. DNA was extracted using the PicoPure DNA extraction kit (Arcturus). Sequencing of cDNA/gDNA was carried out using the 2x Big Dye Terminator v3.1 reagent (Applied Biosystems). Unincorporated dye terminators were removed with the DyeEx 2.0 Spin kit (Qiagen) and the purified products were run on the ABI 3730 DNA analyser (Applied Biosystems). Primers used to screen the hotspot mutation regions in *Apc, Tp53, Ctnnb1* and *Kras* are available on request. PCR products from *Ctnnb1* mutant polyps were TA cloned (Promega) and DNA extracted from individual colonies (Lyse and Go, Thermo Scientific) which were subsequently sequenced.

qRT-PCR on individual crypts

After longitudinally opening the gut and washing with PBS, several 2 mm sections were sliced from the distal end of SB3 and the distal end of colon and incubated in 5 ml media (30 mM EDTA in DMEM without Ca^{2+} and Mg^{2+}, 0.5 mM DTT, 2% RNAlater (Life Technologies)) for 15 min at 37°C. The digested tissue was then transferred to a small bijoux of RNase-free PBS using RNase-free forceps. This bijoux was then shaken vigorously for 30 s to release crypts. The contents of the bijoux were poured onto a small petri dish and observed under a dissection microscope. Individual crypts were selected and transferred to eppendorfs containing RLT buffer using a drawn out glass pipette, ready for subsequent RNA extraction with the RNeasy microkit (Qiagen) according to manufacturer's instructions. Carrier RNA (poly-A RNA) was added to sample lysates to improve recovery of RNA. On-column DNase digestion was carried out following manufacturer's instructions following by cDNA synthesis. 10 individual crypts from SB3 from three R482Q/R482Q, R482Q/+, Fbxw7$^{-/-}$ and wildtype genotypes were isolated.

Absolute quantification qRT-PCR was performed on the ABI 7900HT cycler (Applied Biosystems) for each sample (at least in duplicate, but in triplicate whenever possible) and data were analysed using standard protocols to calculate relative expression with the dCT method described in Livak *et al* (*Methods*, 2001. 25: 402-8), with *Gapdh* serving as an endogenous control. TaqMan probes used Lgr5 (Mm00438890ml).

Array comparative genomic hybridisation

DNA was extracted from a formalin-fixed-paraffin-embedded R482Q/+ tumour using a phenol-choloform DNA extraction method. aCGH was performed by collaborators at the Medical University of Gratz. The Agilent 4x44k platform was used, following manufacturer's instructions. One exception from the protocol was that the BioPrime array CGH genomic labelling system (Invitrogen) was used rather than the Agilent labelling kit. Genomic DNA from a female C57Bl/6J mouse was used as reference DNA.

Alcian-blue stain for goblet cells

Sections were dewaxed in xylene for 5 min, then rehydrated through graded ethanols (100%, 90%, 70%) for 5 min each followed by 2 min in tap H_20. Slides were then stained in alcian-blue solution (Sigma) for 30 min, then washed in running tap H_20 for 2 min, before being rinsed in dH_20. Slides were then stained in nuclear fast red solution (Sigma) for 5 min and washed in running tap H_20 for 1 min. Slides were then dehydrated through degraded alcohols for 2-5 min each, before mounting a coverslip with DPX. The average number of goblets cells per villus was quantified by counting 100 villi from SB3 in at least three mice of each genotype.

Quantification of Enteroendocrine Cells

In morphologically normal intestinal tissue of *Fbxw7*-mutant mice, abundance of enteroendocrine cells was determined by counting the number of Chromogranin-A positive cells in SB3 in 100 villus-crypt units.

Figure S1: Fbxw7^{R482Q} mouse

A: Structural model of FBXW7 protein. The pink residues comprise the F-box domain, blue the WD40 domain, yellow arginines 465, 479 and 505 and brown the CPD sequence of cyclin E. Arginines 465, 479 and 505 are found at the apex of the β-propellor sheets. Images generated using Cn3D software (NCBI).

B: Targeting scheme to generate *Fbxw7*$^{fl(R482Q)/+}$ mouse. Upper panel shows the loxP-flanked allele with regions of homology, loxP sites, genomic exons 9-11 and a repeated exon 9-11 in which a R482Q mutation has been introduced by site-directed mutagenesis. The lower panel represents the allele after Cre-mediated recombination which removes wildtype exons 9-11 and the polyA signal, thus allowing the transcription of the R482Q mutated exon 9. The arrows with corresponding numbers depict the PCR primers used to genotype the R482Q mice.

C: Tissue-specific knock-in of R482Q mutation mediated by *Vill*-Cre recombination in mature R482Q/+ (*Vill*-Cre $^{+}$) and *Fbxw7*$^{fl(R482Q)/+}$ (*Vill*-Cre $^{-}$) mice. Primer pair 1 and 3 show the presence of the R482Q loxP-flanked allele whereas primer pair 1 and 6 show Cre-mediated knock-in of the R482Q mutation.

D: cDNA sequencing confirms expression of the heterozygous mutant allele in R482Q/+ mouse intestines.

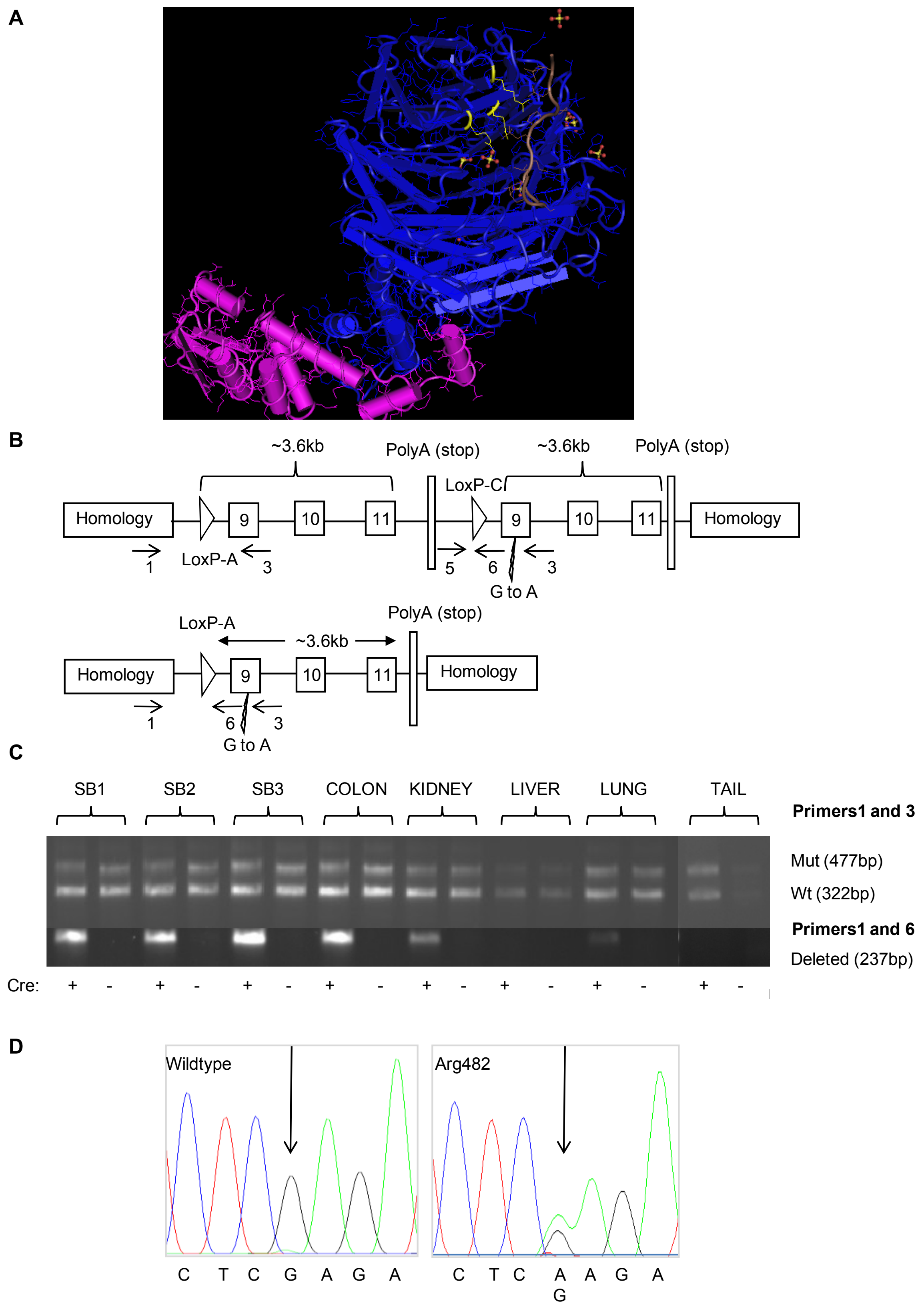
A
B
~3.6kb
PolyA (stop)
~3.6kb
PolyA (stop)
LoxP-C
Homology
9
10
11
9
10
11
Homology
1
LoxP-A
3
5
6
3
G to A
LoxP-A
PolyA (stop)
~3.6kb
Homology
9
10
11
Homology
1
6
3
G to A
C
SB1
SB2
SB3
COLON
KIDNEY
LIVER
LUNG
TAIL
Primers1 and 3
Mut (477bp)
Wt (322bp)
Primers1 and 6
Deleted (237bp)
Cre: + - + - + - + - + - + - + - + -
D
Wildtype
Arg482
C T C G A G A
C T C A A G A
G

Figure S2: Differentiated cell types in the intestines of Fbxw7 mutant and wildtype mice. Alcian blue and fast acid red staining identify goblet cells. Immunohistochemistry-based analysis identifies enteroendocrine (Chromogranin-A), enterocyte (alkaline phosphatase) and Paneth (lysozyme) cells. Scale bars represent 50 μm.

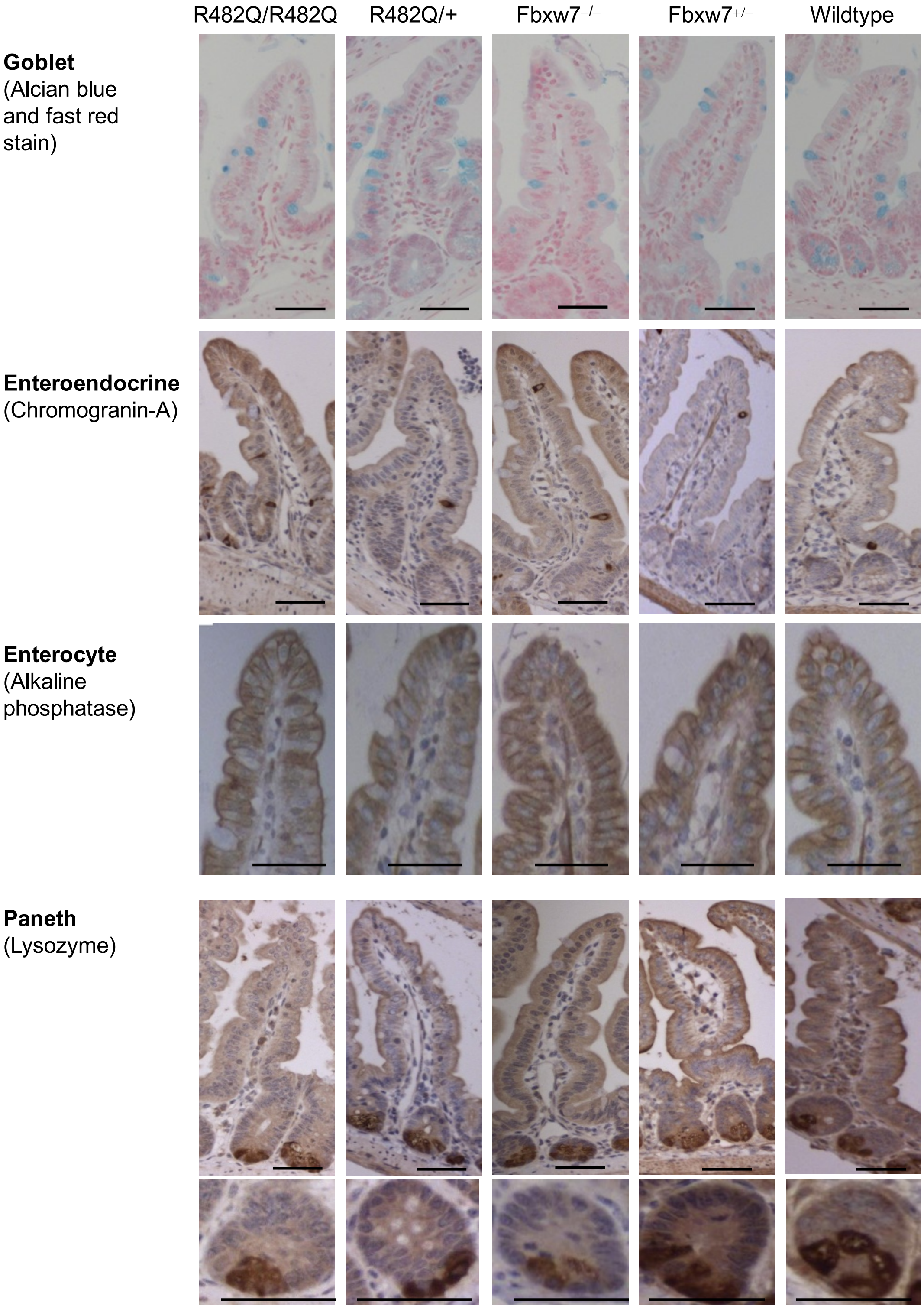

Figure S3: Proliferation and apoptosis analysis in Fbxw7 mutant intestines

A: Scale bars represent 150 µm. Arrowheads indicate Caspase3 positive (apoptotic) cells.

B: The percentage of Ki67-positive (proliferative) cells per crypt was assessed in 200 crypts in SB3, for at least 3 mice for each genotype (p>0.05 in all cases, Student t-test).

C: The mean number of Caspase3-positive cells per crypt-villus unit in SB1, SB2 and SB3 in 100 crypt-villus units were assessed in at least 3 mice of each genotype. Error bars represent the SEM.

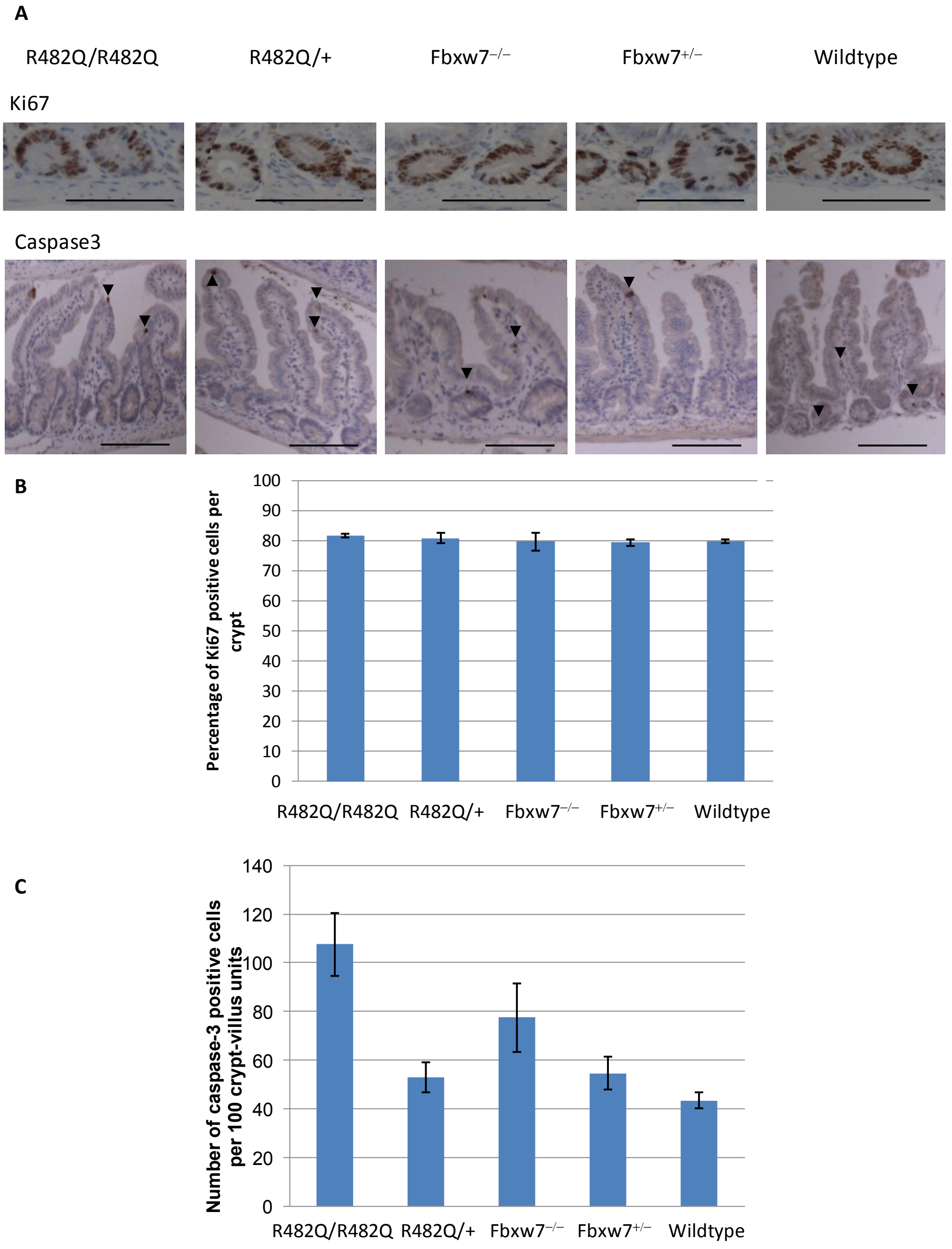

Figure S4: Investigation of multiple *Fbxw7* substrates in normal intestinal tissue

Expression of Fbxw7 target proteins in whole intestines from *Fbxw7*-mutant 6-week old mice was assessed using western analysis in two technical replicates with β-actin as a loading control. Illustrative blot results are shown and the bar chart shows quantification (test:control ratio of normalised pixel intensity shown relative to that of wildtype mice). We compared substrate levels in R482Q/+, R482Q/R482Q, *Fbxw7*$^{+/-}$ and *Fbxw7*$^{-/-}$ mice with wildtype animals. The only significant differences were elevated levels of cyclin E in R482Q/R482Q and *Fbxw7*$^{-/-}$ mice (*p*=0.003 and *p*=0.025 respectively, t-test). We found significant trends in substrate levels for cyclin E and Nicd1 across wildtype, R482Q/+ and R482Q/R482Q animals (*p*=0.021 and *p*=0.011 respectively, trend test). Finally, we tested whether there were differences between the following genotypes: R482Q/+ and *Fbxw7*$^{+/}$; R482Q/+ and *Fbxw7*$^{-/}$; R482Q/R482Q and *Fbxw7*$^{-/-}$. Nicd1 was more highly expressed in *Fbxw7*$^{-/-}$ than R482Q/R482Q tissues (*p*=0.016, t-test). c-Myc was also more highly expressed in *Fbxw7*$^{-/-}$ than R482Q/R482Q (*p*=0.042, t-test), but the levels of c-Myc varied greatly, and inconsistently, between samples.

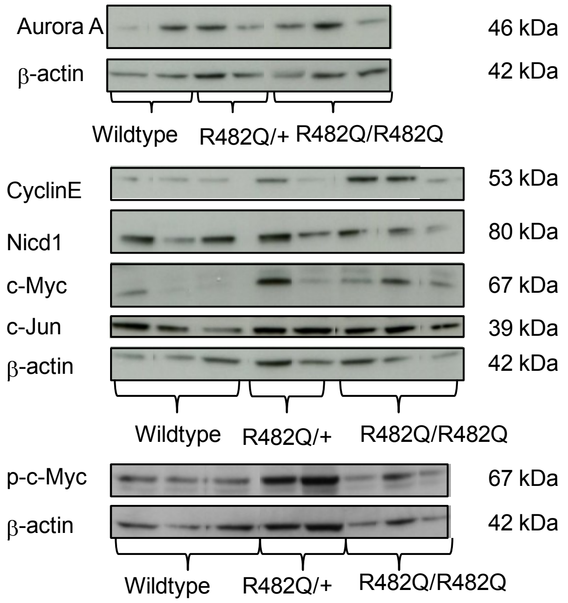

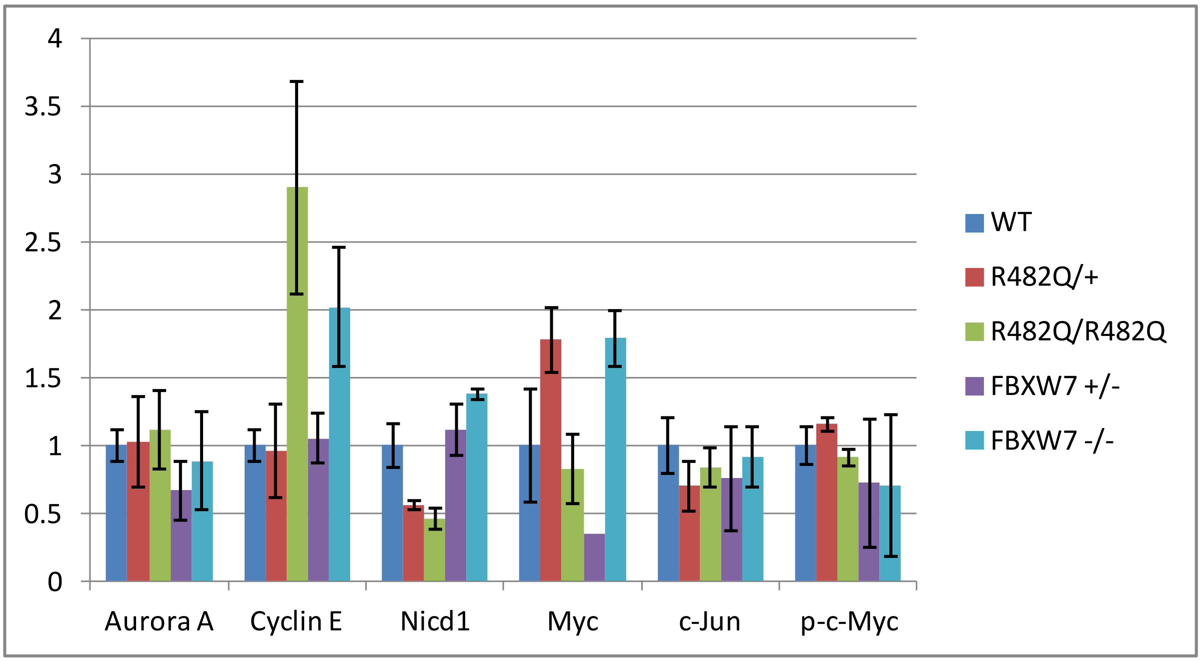

Figure S5: Adenocarcinoma in the small intestine of a R482Q/+ mouse

A: Gross appearance of tumour

B: Methylene blue stained tumour

C-D: H and E stained sections of tumour showing invasion into the muscle layer (dotted circle). Scale bars represent 150 μm.

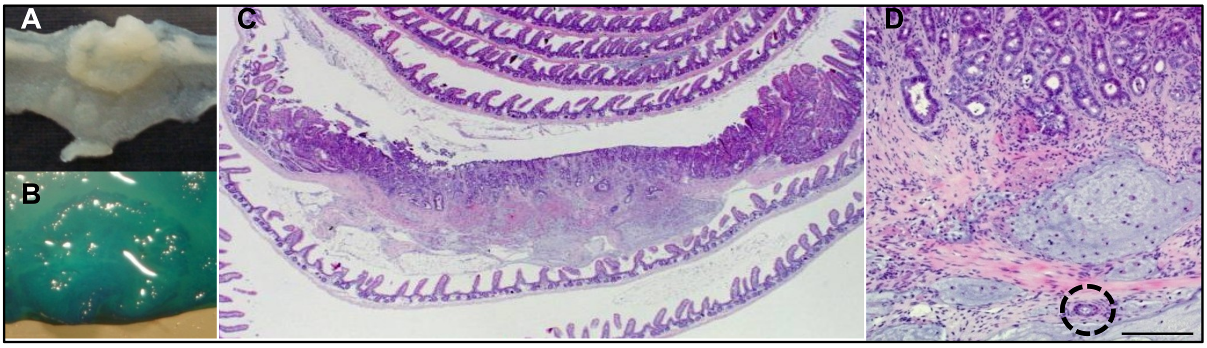

Figure S6: *Ctnnb1* mutations in R482Q/+ polyps

DNA from polyps was laser capture microdissected. Corresponding normal intestinal epithelium from the same animal was used as a control. Sequencing traces were analysed using the ApE programme. Note the lower intensity of mutant chromatograms in comparison to wildtype, suggesting the mutations were not present in all tumour cells indicating polyclonality of the tumours. To eliminate the possibility of the mutant traces being spurious background sequencing, PCR products were TA cloned and individual colonies screened. The mutant *Ctnnb1* trace was identified in 29% of single colonies (n=49).

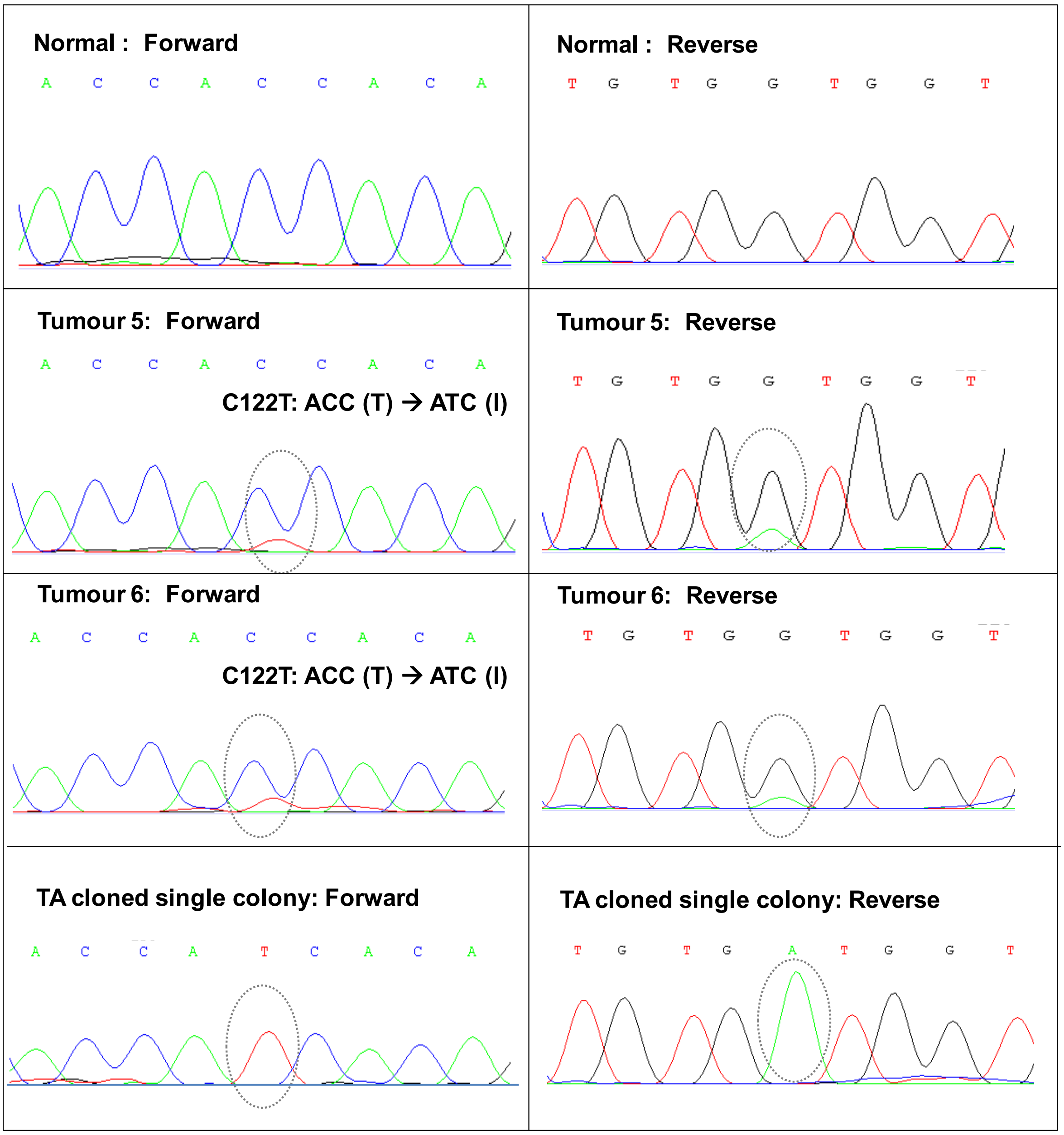

Figure S7: R482Q mutation enhances tumorigenesis in R482Q/+; *Apc* compound-mutant mice

Nine age- and litter-matched R482Q/+ Min mice and Min mice were analysed. Both test and control mice within each litter were collected when the former showed signs of phenotype. Nine Fbxw7$^{+/-}$ Min, 13 R482Q/+ 1322T mice and 5 Fbxw7$^{+/-}$ 1322T were analysed similarly alongside the appropriate controls. As only very few colon polyps were identified in both test and control samples these were excluded from analysis. Since symptoms of polyp burden depend on both tumour numbers and size, whilst 1322T compound mice have more severe polyposis, this paradoxically results in fewer polyps at presentation, because the polyps are larger than in Min mice.

A: Box and whiskers plot of the polyp burden in the small intestines. The line within the box represents the median, lines at the ends of the box represent 25th and 75th centiles and lines at the end of the whiskers represent 5th and 95th centiles.

B: Proportion of polyps in each intestinal location,

C: Proportion of polyps in each size category.

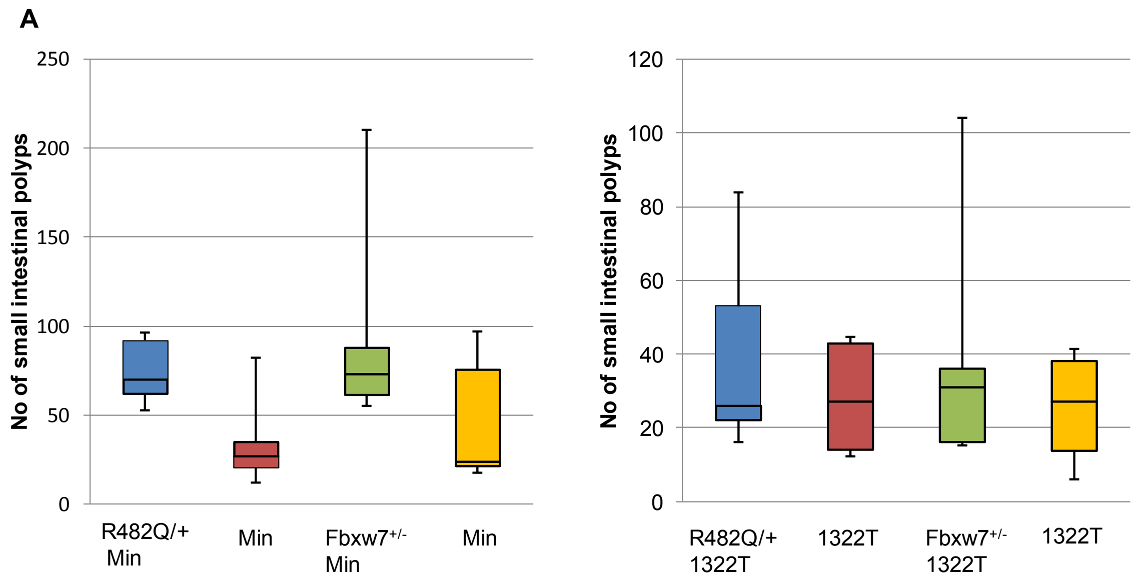

B

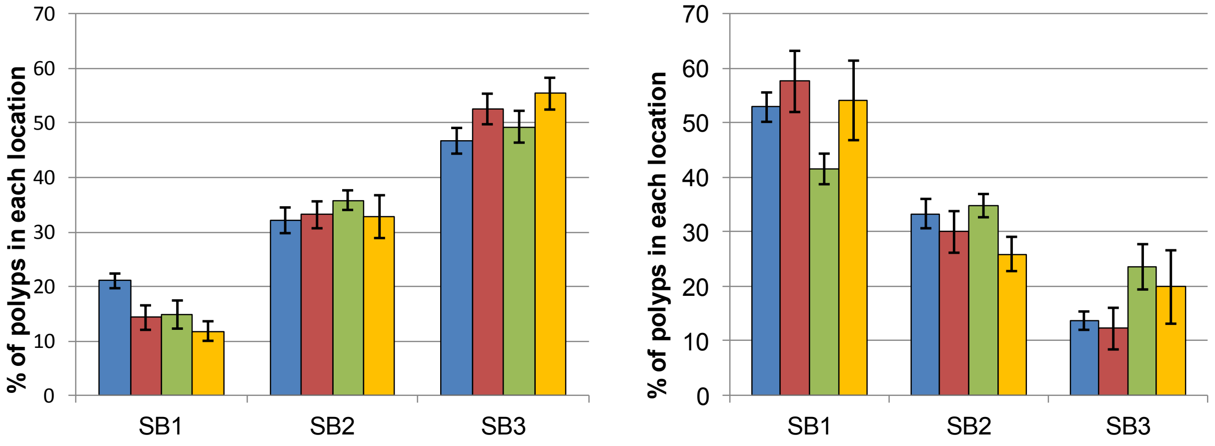

% of polyps in each location
0
10
20
30
40
50
60
70
SB1
SB2
SB3
% of polyps in each location
0
10
20
30
40
50
60
70
SB1
SB2
SB3

C

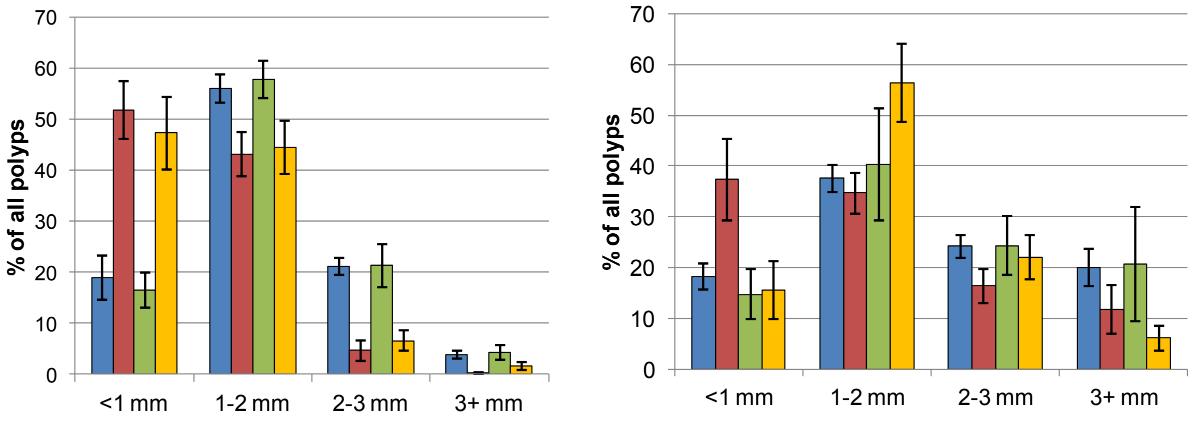

% of all polyps
0
10
20
30
40
50
60
70
<1 mm
1-2 mm
2-3 mm
3+ mm
% of all polyps
0
10
20
30
40
50
60
70
<1 mm
1-2 mm
2-3 mm
3+ mm

Figure S8: No significant difference in the levels of Fbxw7 substrates in R482Q/+; 1322T polyps

β-actin was used as a loading control. Western blots were all repeated in at least two technical replicates. Other analyses are as in Figure S4. See Figure 3 for Tgif1 and Klf5 data. For all comparisons shown here, $p>0.05$ (t-test).

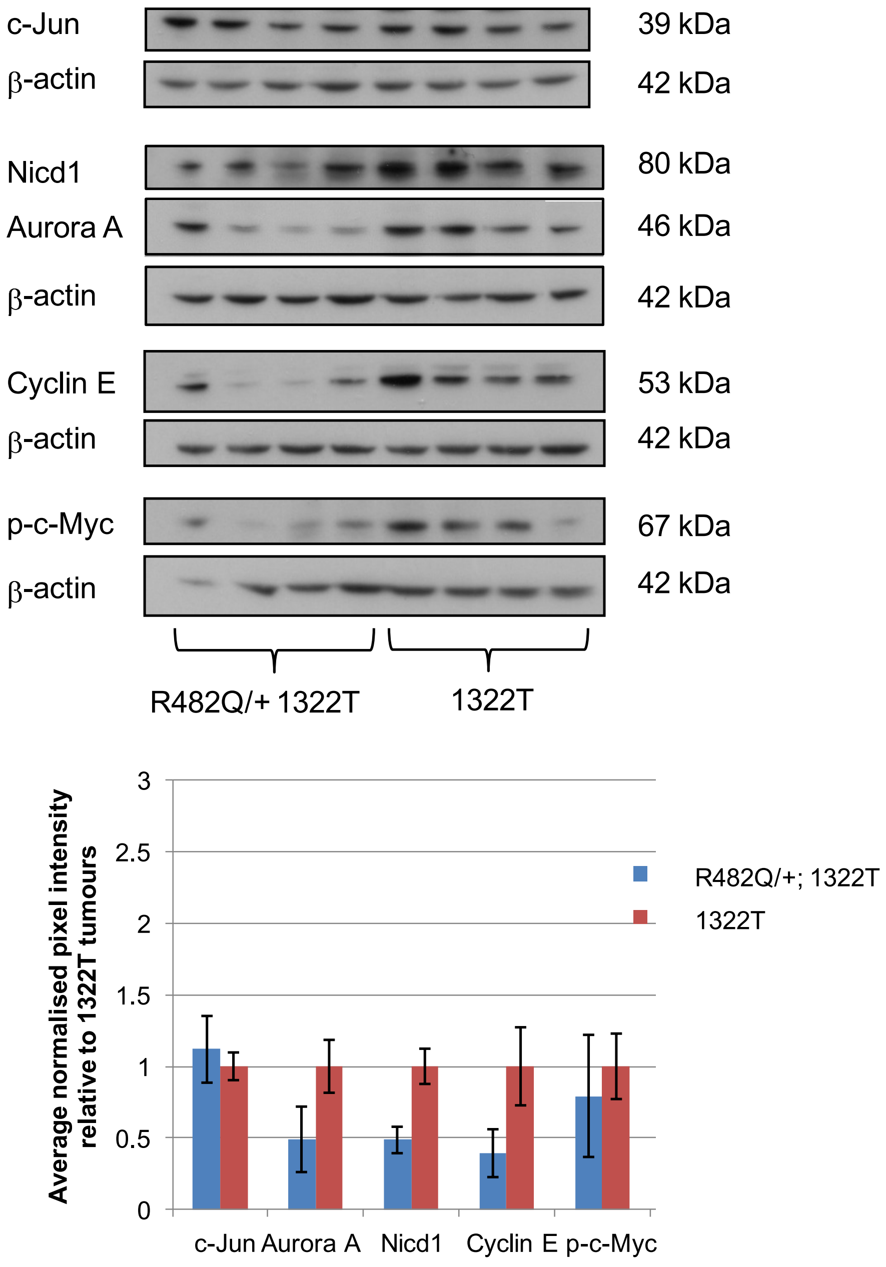

Supplement: Web supplement [file gutjnl-2013-304719-s1.pdf]
